# The Role of Neoadjuvant Chemotherapy in Patients With Advanced Endometrial Cancer at King Abdulaziz Medical City (KAMC), Saudi Arabia From 2010 to 2022

**DOI:** 10.7759/cureus.60752

**Published:** 2024-05-21

**Authors:** Abdullah F Tayeb, Fahad S Subahi, Ahmad Z Al-Ghanmi, Abdulrahman A Zehairy, Abdullah S Alyamani, Abdulaziz A Kano, Hatim Al-Jifree, Mawaddah Alahmadi, Syed S Aga, Wala Mehros

**Affiliations:** 1 College of Medicine, King Saud bin Abdulaziz University for Health Sciences, Jeddah, SAU; 2 Medicine, King Abdullah International Medical Research Center, Jeddah, SAU; 3 Oncology, King Abdulaziz Medical City, Ministry of National Guard Health Affairs, Jeddah, SAU; 4 King Abdullah International Medical Research Center, Medicine, Jeddah, SAU; 5 Obstetrics and Gynaecology, College of Medicine, King Saud bin Abdulaziz University for Health Sciences, Jeddah, SAU; 6 Oncology, King Abdullah International Medical Research Center, Jeddah, SAU; 7 Department of Basic Medical Sciences, King Saud bin Abdulaziz University for Health Sciences, Jeddah, SAU; 8 Medicine, King Abdulaziz Medical City, Ministry of National Guard Health Affairs, Jeddah, SAU

**Keywords:** neoadjuvant chemotherapy (nact), chemotherapy, advanced stages of tumor, endometrial cancer prognosis, gynecology-oncology, endometrial cancer

## Abstract

Background: Endometrial cancer (EC) has multiple modalities of treatment including neoadjuvant chemotherapy (NACT). There is limited research work conducted in Saudi Arabia that shows the benefits of using NACT, followed by interval debulking surgery (IDS) for stages III-IV EC patients. Hence, this study aims to evaluate the effectiveness of using NACT compared to other modalities of treatment in the last 11 years in Saudi Arabia.

Methods: The data of the patients were collected retrospectively between 2010 and 2022 at Princess Noura Oncology Centre, Jeddah, Saudi Arabia. The population was divided based on receiving NACT or taking other modalities for the purpose of assessing the mean survival time in both groups. Best-case and worst-case scenario models were used to illustrate the survival rate of both stages.

Results: Forty patients with stages III-IV EC were included and grouped based on the treatment modality. Fourteen (35%) patients were receiving NACT followed by IDS compared with 26 (65%) patients who were using other modalities. In both stages III-IV patients, the mean survival time in the best-case scenario was 49 months in patients treated with NACT, and 82 months in patients who received other modalities. Regarding the worst-case scenario, the average survival time for patients treated with NACT was 22.89 months, which was significantly lower than the average survival time of 56.30 months for patients treated with other therapies.

Conclusion: In the worst-case scenario, advanced EC patients who underwent NACT had a lower mean survival time than other treatment modalities. However, using NACT is not connected to the outcome in the best-case scenario.

## Introduction

Endometrial cancer (EC) is the most frequent gynecological malignancy around the globe, and it is becoming more common in affluent countries [1]. Although the prognosis of EC strongly depends on the stage of the disease, it has a good prognosis for patients diagnosed in the early stages and a poor prognosis for patients diagnosed with advanced stages [2]. In Saudi Arabia, cancer has been increasing over the last 40 years because of the dramatic socioeconomic status changes, which led to changes in people's lifestyles [3]. According to a study that was done in 2018 about the changes in the incidence of cancer in Saudi Arabia between 1990 and 2016, uterine cancer showed an increase in new cases, from 44 to 450 cases. Also, the mortality rate had been increasing from approximately 5% in 1990 to 13% in 2016. Gynecological malignancies are the most common type of cancer that affects Saudi women [3]. According to the Saudi Cancer Registry in 2018, EC was the fourth most incidence among Saudi females with 564 cases, representing 6.4% of all malignancies among Saudi females, and 3.6% in all Saudi nationals diagnosed with cancer [4].

Neoadjuvant chemotherapy (NACT) was initially used to treat locally advanced breast cancer; however, it is now used for multiple types and stages of cancer including advanced EC [5]. NACT’s main objective is to shrink the cancerous tumor which facilitates the removal in the surgery or makes inoperable tumors operable. It also has other benefits such as acknowledging the response of cancer tumors to specific medications and increasing overall survival (OS) and disease-free survival (DFS) [5]. Furthermore, patients treated with NACT have shown a high survival rate among different advanced cancer types. Treatment with NACT followed by interval debulking surgery (IDS) for patients diagnosed with advanced epithelial ovarian cancer had shown lower side effects and higher survival rates than primary debulking surgery alone. Moreover, NACT has started to become a standard option of treatment for patients diagnosed with advanced epithelial ovarian cancer [6]. On the other hand, a cohort study was conducted by Claire et al. about the association of NACT with OS in metastatic EC patients. The study included 952 women who were treated with NACT. It was found that using NACT decreases the mortality rate only in the first few months, followed by increased mortality in a long-term manner [7]. According to statistics, patients who received NACT had significantly shorter lengths of operations and time of hospitalizations in comparison with the other options of treatment [8,9].

However, there is limited research work conducted in Saudi Arabia that shows the benefits of using NACT, followed by IDS for stages III-IV EC patients. Hence, this study aims to evaluate the effectiveness of using NACT compared to other modalities of treatment in the last 11 years at Princess Noura Oncology Center, Jeddah. Our main objectives are assessing the rate of response to receiving NACT in EC and comparing the OS in stage III and IV groups.

## Materials and methods

This research was a retrospective cohort study design. The study was conducted at Princess Noura Oncology Center located in King Abdulaziz Medical City, Jeddah, Saudi Arabia. Moreover, the facility was a well-equipped tertiary care center that takes part in the care of cancer patients in the western region of Saudi Arabia. Institutional Review Board approval was obtained and reference number JED-22-427780-47006 was issued on the 12th of April 2022. The inclusion criteria consist of all histopathological subtypes of EC, International Federation of Gynecology and Obstetrics (FIGO) stages III-IV, and receiving NACT followed by IDS. Exclusion criteria consist of the presence of another primary cancer in less than 12 months, early-stage EC FIGO stage I-II, recurrent EC, and any treatment other than chemotherapy.

The sampling technique was consecutive sampling which included all populations of EC patients (FIGO stages III and IV) who met the criteria from January 2010 to December 2022, with a sample size of 40 patients divided into two groups. Fourteen patients were included in group 1, which constitutes the group who received the NACT. Group 2, the control group, included 26 patients who received all other treatment modalities e.g. chemotherapy alone or hormonal thereby, with a confidence interval of 95% and power of 80%.

The data collection sheet was used to collect demographic data, diagnosis date, presence of comorbidities, beginning of treatment date, tumor size, lymph nodes involvement, type of chemotherapy whether adjuvant or neoadjuvant, type of surgery, complications, and progression-free survival. The medical files were assessed through the medical department in all selected patients electronically on BestCare 2.0 (ezCaretech, Seoul, South Korea) if the patient presented after 2016 or through paper form if the patient presented between 2010 and 2016.

Data analysis was done by Statistical Package for the Social Sciences (IBM SPSS Statistics for Windows, IBM Corp., Version 29.0, Armonk, NY). Mean and standard deviation were used to describe the numerical data, e.g. age and BMI. Frequencies and percentages were utilized to describe the categorical data, e.g. comorbidities, FIGO stage, and histological subtype. Survival rates were analyzed by Kaplan-Meier curves, and through the log-rank test, and a p-value less than 0.05 was considered statistically significant. Best-case and worst-case scenario models were used to handle the effect of the lost to follow-up patients. In the best-case scenario, we considered the lost to follow-up patients alive along the follow-up, while in the worst-case scenario, the date of last contact was considered the date of death. Furthermore, sub-stratified analysis was used to observe the differences in the survival curves with respect to the FIGO stages of the patients.

## Results

The age of diagnosis, body mass index (BMI), marital status, and comorbidities of the patients are illustrated in Table 1, which were assessed by the medical records at Princess Noura Oncology Center. The age of the patients (n=40) at diagnosis ranged from 29 to 89 years, with an average of 59.9 (SD=13.3) with a mean BMI of 29.9 (SD=5.7). In relation to marital status, 75% were married, with a total number of 30 patients. Regarding comorbidities, 25 of the patients were hypertensive, and only nine patients were obese. The number of patients who have endometrioid adenocarcinoma was the highest compared with other subtypes, with a total number of 18, which was followed by serous papillary adenocarcinoma, mixed carcinoma, and undifferentiated carcinoma respectively, as shown in Table 2. The majority of the patients had stage IIIA with 30% (n=12), stage IIIC1 with 22.5% (n=9), and stage IVB with 22.5% (n=9) as well. The number of patients who underwent NACT in both groups was 14 compared to 26 patients with different options of treatment. There were 28 patients with stage III, and nine of those underwent NACT. Regarding stage IV patients, there were 12 patients, including five patients who were treated with NACT.

**Table 1:** Main characteristics of the study population

	Cases (n=40)	Mean	Std. Deviation
BMI	40	29.89	5.703
Age at the diagnosis	40	59.88	13.311
	Cases (n=40)	Percent	
Married	30	75	
Not married	3	7.5	
Divorced	2	5	
Widow	4	10	
Unknown	1	2.5	
	Cases (n=40)	Percent	
Hypertension	25	62.5	
Diabetes mellitus	23	57.5	
Obesity	9	22.5	

**Table 2:** EC histological subtypes, stages, and modality of treatment for stages III and IV patients EC: endometrial cancer; NACT: neoadjuvant chemotherapy

	Cases (n=40)	Percent	
Endometroid Adenocarcinoma (EA)	18	45	
Serous Papillary Adenocarcinoma (SPA)	12	30	
Mixed Carcinoma (MC)	6	15	
Undifferentiated Carcinoma (UC)	2	5	
EA + SPA	1	2.5	
EA + SPA + MC	1	2.5	
	Cases (n=40)	Percent	
IIIA	12	30	
IIIB	5	12.5	
IIIC1	9	22.5	
IIIC2	2	5	
IVA	3	7.5	
IVB	9	22.5	
	Treated with NACT	Total N	
Stage III	Yes	9	
	No	19	
	Overall	28	
Stage IV	Yes	5	
	No	7	
	Overall	12	
Overall	Overall	40	

**Figure 1:**
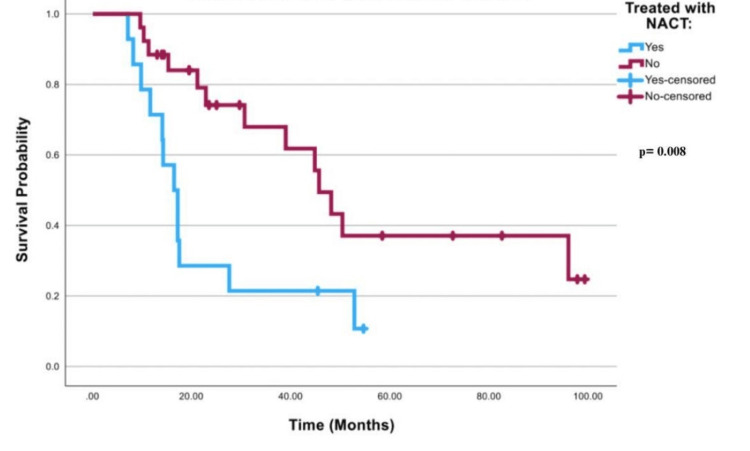
Survival curve for all patients in the worst-case scenario NACT: neoadjuvant chemotherapy

**Figure 2:**
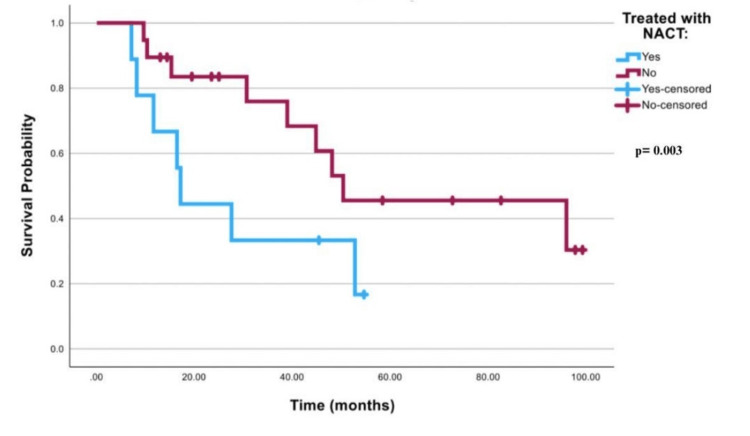
Survival curve for stage III patients only in the worst-case scenario NACT: neoadjuvant chemotherapy

**Figure 3:**
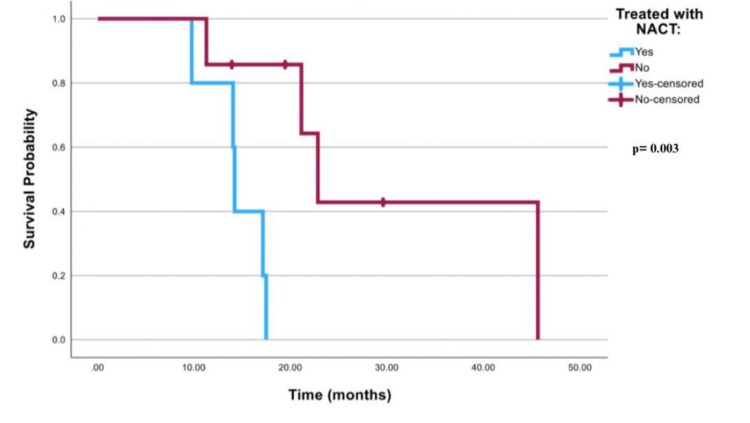
Survival curve for stage IV patients only in the worst-case scenario NACT: neoadjuvant chemotherapy

Figure 1 shows the survival curve for all worst-case scenario patients that was significant with a p-value of 0.008. In Table 3, it is illustrated that patients treated with NACT had a mean survival time of 22.89 months (95% CI; 14.13-31.65), while patients treated with other modalities had a mean of 56.30 months (95% CI; 40.97-71.63). Table 4 illustrates that FIGO stage III patients who were treated with NACT had a mean of 27.58 months (95% CI; 15.02-40.14). FIGO stage 3 patients who were treated with other modalities had a mean of 62.71 months (95% CI; 45.35-80.06). FIGO stage 4 patients who were treated with NACT had a mean of 14.45 months (95% CI; 11.74-17.17), while patients who were treated with other modalities had a mean of 30.5 months (95% CI; 17.19-43.81). Log rank for worst-case scenario FIGO stages III or IV cancer was 0.003, as shown in Figures 2-3.

**Table 3:** Mean survival times in stages III and IV combined in the worst-case scenario NACT: neoadjuvant chemotherapy

	Mean			
			95% Confidence Interval	
Treated with NACT	Estimate	Std. Error	Lower Bound	Upper Bound
Yes	22.897	4.470	14.134	31.659
No	56.302	7.821	40.973	71.631
	Chi-Square	df	Sig.	
Log Rank (Mantel-Cox)	6.980	1	.008	

**Table 4:** Mean survival times in stages III and IV the worst-case scenario NACT: neoadjuvant chemotherapy

		Mean				
				95% Confidence Interval		
Stage III or IV	Treated with NACT	Estimate	Std. Error	Lower Bound	Upper Bound	
Stage III	Yes	27.586	6.407	15.029	40.143	
	No	62.710	8.855	45.354	80.066	
Stage IV	Yes	14.456	1.386	11.740	17.172	
	No	30.507	6.792	17.194	43.819	
	Chi-Square	df		Sig.		
Log Rank (Mantel-Cox)	8.615	1		.003		

**Figure 4:**
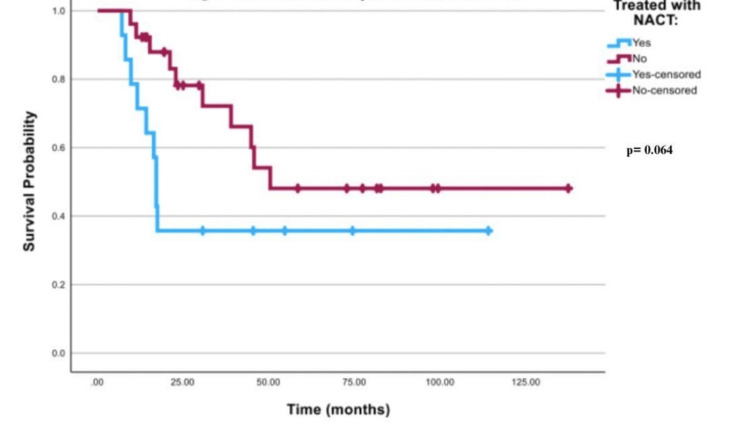
Survival curve for all patients in the best-case scenario NACT: neoadjuvant chemotherapy

**Figure 5:**
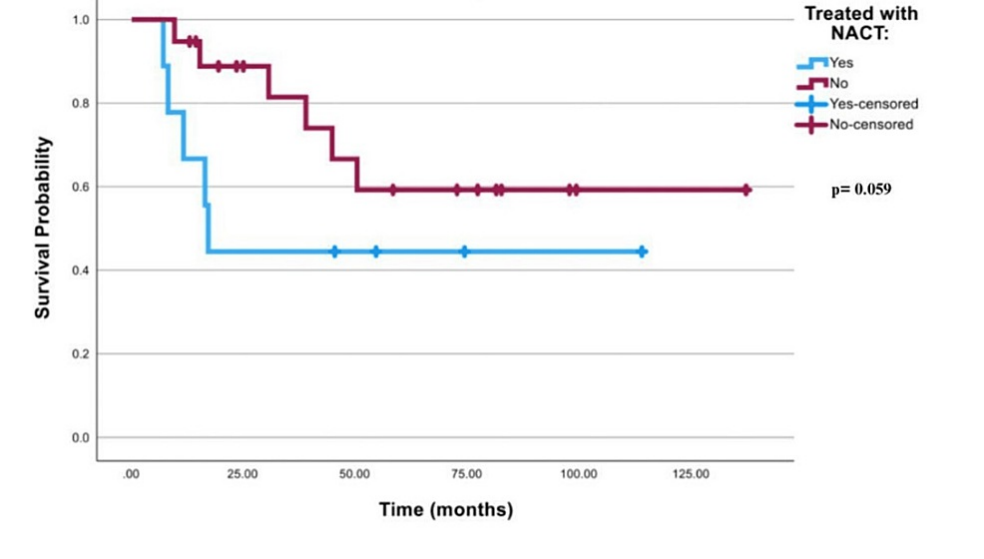
Survival curve for stage III patients only in the best-case scenario NACT: neoadjuvant chemotherapy

**Figure 6:**
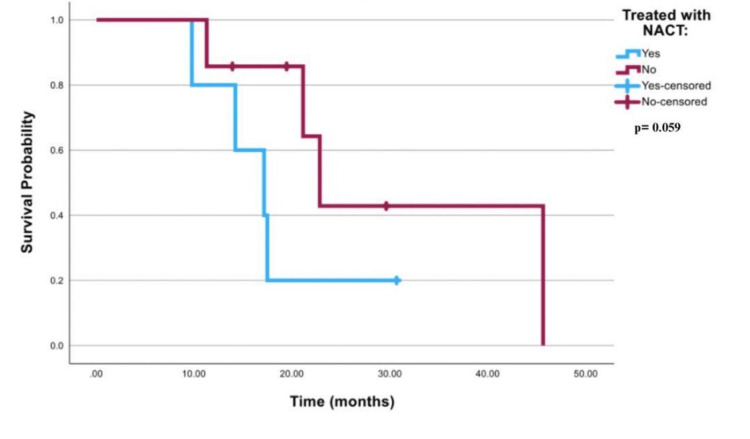
Survival curve for stage IV patients only in the best-case scenario NACT: neoadjuvant chemotherapy

The survival curve that is demonstrated in Figure 4 for all best-case scenario patients was insignificant with a p-value of 0.064. In Table 5, mean survival time in both FIGO stage III and stage IV patients in the best-case scenario was 49 months (95% CI; 23.78-74.38) in patients treated with NACT, and 82 months (95% CI; 57.73-106.50) in patients who received other treatment modalities. Table 6 shows that the mean survival time was 57.2 months (95% CI; 24.14-90.317) in best-case scenario FIGO stage III patients who received NACT vs 94.7 months (95% CI; 68.16-121.24) for stage III best-case scenario patients who received other treatment modalities. Mean survival time was 17.8 months (95% CI; 11.67-23.89) in FIGO stage IV best-case scenario patients treated with NACT vs 30.5 months (95% CI; 17.19-43.81) for stage IV best-case scenario patients who received other treatment modalities. Log-rank for best-case scenario FIGO stages III or IV cancer was 0.59, as illustrated in Figures 5-6.

**Table 5:** Mean survival times in stages III and IV combined in the best-case scenario NACT: neoadjuvant chemotherapy

	Mean			
			95% Confidence Interval	
Treated with NACT	Estimate	Std. Error	Lower Bound	Upper Bound
Yes	49.082	12.908	23.781	74.382
No	82.115	12.439	57.734	106.496
	Chi-Square	df	Sig.	
Log Rank (Mantel-Cox)	3.418	1	.064	

**Table 6:** Mean survival times in stages III and IV in the best-case scenario NACT: neoadjuvant chemotherapy

		Mean				
				95% Confidence Interval		
Stage III or IV	Treated with NACT	Estimate	Std. Error	Lower Bound	Upper Bound	
Stage III	Yes	57.233	16.880	24.148	90.317	
	No	94.698	13.542	68.156	121.240	
Stage IV	Yes	17.777	3.118	11.665	23.889	
	No	30.507	6.792	17.194	43.819	
	Chi-Square	df		Sig.		
Log Rank (Mantel-Cox)	3.578	1		.059		

## Discussion

Summary of main results

A best-case scenario comparison of the survival rates between advanced EC patients who were treated with NACT and other modalities had shown that NACT patients had a lower survival rate than patients who were treated by other modalities, with or without separating them by the stages. However, these results are considered statically insignificant, i.e. using NACT is not associated with the outcome of the patient in the best-case scenario. While in the worst-case scenario, the comparison between advanced EC patients who were treated with NACT and other modalities showed that patients treated with other modalities had significantly higher mean survival time than patients treated with NACT, with or without separating them by the stages. As a result, administering NACT to advanced EC patients can lower their mean survival time.

Results in the context of published literature

The NACT usage in treating advanced EC patients is still under debate. However, the present study illustrates that the usage of NACT followed by IDS had a lower survival rate in both best-case and worst-case scenarios. Our findings are also somewhat similar, regarding the OS, to a multicenter study that was considered to evaluate the prognosis of initial treatment in 426 patients with stage IVb. The median OS for 279 patients who underwent primary surgery was better than both 125 patients treated with primary chemotherapy and 22 patients treated with palliative chemotherapy. It was shown that the OS in patients who underwent primary surgery was 21 months compared to 12 months in patients treated with primary chemotherapy, whether they underwent surgery or not [10]. In another study that included 4890 women with metastatic EC, and 952 women treated with NACT, they found that patients who were treated with NACT had a decrease in the mortality rate in the first eight months; however, after that, survival curves crossed, and the receipt of NACT had higher mortality. The study concluded that women who were treated with NACT had superior survival in the short term, while patients who underwent IDS had a more favorable long-term prognosis [7].

A systematic review, that was conducted recently by Capozzi et al., included 21 studies regarding the optimal management of stage IVB EC, 10 of which were on NACT [11]. Seven of them were in line with our findings, and their results were either that NACT has no difference in the OS, DFS, or disease-specific survival (DSS), or it increases the mortality rate in the long-term prognosis. The review concluded by stating the presence of the NACT option and the possibility of its suitability only for serous histology with a low level of evidence.

Nevertheless, several other studies addressed the benefits of NACT followed by IDS. Khouri et al. illustrated the role of NACT in patients with advanced stages of EC. They identified 39 patients with advanced-stage EC who were contraindicated to primary surgery due to unresectable disease, poor performance, or both. Forty-one percent (n=16) of the patients who underwent NACT and IDS compared to 59% (n=23) who underwent NACT alone. The patients who had IDS and NACT had 81% optimal cytoreduction and disease status after NACT completion 56% partial response, 3% stable disease, and 41% progression of the disease. Overall, both responses to NACT and IDS were associated with improved survival [12]. Additionally, De Lange et al. investigated the treatment strategies in advanced EC patients. The study included 102 patients with serious, endometroid, clear cell, and other histological subtypes; however, only 78% (n=80) of the patients underwent NACT followed by IDS, whether they had adjuvant chemotherapy later or not. The outcome of IDS following NACT in the patients was complete in 60% (n=48), optimal in 28.9% (n=23), and incomplete in 11.3% (n=9) of cases. Moreover, the median OS rate was 27 months. It was concluded that the efficacy of this treatment strategy is appropriate for patients who had obstacles in performing primary surgery [13].

Strengths and weaknesses

Although our study was carried out by analyzing data from Princess Noura Oncology Center, we acknowledge several important limitations. First, the main obstacle in the research was the small sample size. However, we attempted to minimize the effect of it by conducting a consecutive sampling technique that covers a long time span from 2010 to 2022. Furthermore, we analyzed the data using a sub-stratified analysis to assess and mitigate the effect of the small sample size. Another possible obstacle is the lost to follow-up with the patients. In this study, 15% of the samples were lost to follow-up cases; however, the best-case/worst-case scenario model has been used to ensure more accurate outcomes.

Implications for practice and future research

As previously mentioned, there is limited research work regarding the usage of NACT in patients with advanced EC. In this research, we tried to illustrate the controversy in that field, investigate the rate of response to receiving NACT, and determine the treatment efficacy. Furthermore, we contributed our outcome experience in Princess Noura Oncology Center to the public which might affect physicians' decisions in the future.

## Conclusions

In summary, using NACT for advanced EC patients can lower their mean survival time in the worst-case scenario. While in the best-case scenario administering NACT is not associated with the outcome of the patient. Further studies are warranted to observe the role of NACT on each FIGO stage or histology type separately with the optimal cytoreduction of EC. Experimental studies with fixed variables and a control group may be more beneficial than the currently available observational studies.
